# An Evaluation of Training with an Auditory P300 Brain-Computer Interface for the Japanese Hiragana Syllabary

**DOI:** 10.3389/fnins.2016.00446

**Published:** 2016-09-30

**Authors:** Sebastian Halder, Kouji Takano, Hiroki Ora, Akinari Onishi, Kota Utsumi, Kenji Kansaku

**Affiliations:** ^1^Systems Neuroscience Section, Department of Rehabilitation for Brain Functions, Research Institute of National Rehabilitation Center for Persons with DisabilitiesTokorozawa, Japan; ^2^Department of Psychology I, Institute of Psychology, University of WürzburgWürzburg, Germany; ^3^Brain Science Inspired Life Support Research Center, University of Electro-CommunicationsChofu, Japan; ^4^Department of Neurology, Brain Research Institute, Niigata UniversityNiigata, Japan

**Keywords:** assistive technology, electroencephalography, event-related potentials, P300, auditory stimulation, brain-computer interface, gaze independence

## Abstract

Gaze-independent brain-computer interfaces (BCIs) are a possible communication channel for persons with paralysis. We investigated if it is possible to use auditory stimuli to create a BCI for the Japanese Hiragana syllabary, which has 46 Hiragana characters. Additionally, we investigated if training has an effect on accuracy despite the high amount of different stimuli involved. Able-bodied participants (*N* = 6) were asked to select 25 syllables (out of fifty possible choices) using a two step procedure: First the consonant (ten choices) and then the vowel (five choices). This was repeated on 3 separate days. Additionally, a person with spinal cord injury (SCI) participated in the experiment. Four out of six healthy participants reached Hiragana syllable accuracies above 70% and the information transfer rate increased from 1.7 bits/min in the first session to 3.2 bits/min in the third session. The accuracy of the participant with SCI increased from 12% (0.2 bits/min) to 56% (2 bits/min) in session three. Reliable selections from a 10 × 5 matrix using auditory stimuli were possible and performance is increased by training. We were able to show that auditory P300 BCIs can be used for communication with up to fifty symbols. This enables the use of the technology of auditory P300 BCIs with a variety of applications.

## Introduction

For persons with paresis, such as patients with amyotrophic lateral sclerosis (ALS) or brain injuries, brain-computer interface (BCI) can provide assistive technology for controlling various applications (Birbaumer et al., 1999; McCane et al., 2015). These applications include complex scenarios such as the control of exo-skeletons or basic binary yes/no communication (Sakurada et al., 2013; Hill et al., 2014). Neurodegenerative diseases or brainstem lesions may lead to the locked-in state (LIS) or complete locked-in state (CLIS) in which consciousness is preserved but the person is completely paralyzed (Hayashi and Kato, 1989; Kotchoubey et al., 2003). Such diseases that lead to the CLIS also affect gaze control (Mizutani et al., 1992; Frohman et al., 2005). Furthermore, under certain conditions controlling a BCI with gaze has been shown to induce higher workload than using an eye-tracker in end-users with ALS (Pasqualotto et al., 2015). This calls for a possibility of gaze independent communication and led to the development of auditory BCI (and other gaze independent BCI; Riccio et al. (2012)). These are derived from visual BCI using the P300 event-related potential (ERP) component that can be measured with electroencephalogram (EEG) as first described in Farwell and Donchin (1988). Their design can range from simple but reliable binary choice communication systems (Hill et al., 2005, 2014; Halder et al., 2010) to more complex spelling systems that require higher attentional demands (Furdea et al., 2009; Klobassa et al., 2009; Schreuder et al., 2010; Kleih et al., 2015; Rutkowski and Mori, 2015). Many of the improvements of the underlying paradigms have been achieved by optimizing stimuli material and paradigms. For example based on the design for stereo headphones and spatial features proposed in Käthner et al. (2013), performance has increased considerably by improving stimuli material in Simon et al. (2015) and adding training in Baykara et al. (2016).

In this study we investigated whether training leads to an increase in performance over several sessions with a large number of different stimuli and possible selections. In particular we investigated the case of a BCI system for communication with the Japanese Hiragana syllabary. The syllabary contains 46 symbols and commonly a two-step procedure is used in auditory and visual BCI paradigms to make selections from a matrix of this size (Madarame et al., 2008; Chang et al., 2014; Ikegami et al., 2014). The design used in this study also foresees a two-step selection procedure (as in the aforementioned previous study) with stimuli presented over conventional stereo headphones integrated into an online BCI framework and several sessions of training. We used a set of 15 stimuli for selections from five rows and ten columns. To our knowledge, this is the first time the full Hiragana syllabary was presented for selection from one matrix and one of the largest matrices used in an auditory P300 BCI [Madarame et al. (2008) used a three step approach with one out of five selections and Chang et al. (2014) a five by five matrix, other approaches focused on the symbols of the Latin alphabet]. Three sessions on separate days were conducted and stimuli were presented over stereo headphones. The study was conducted with six persons without impairments and one person with spinal cord injury (SCI).

## Methods

**Figure 1:**
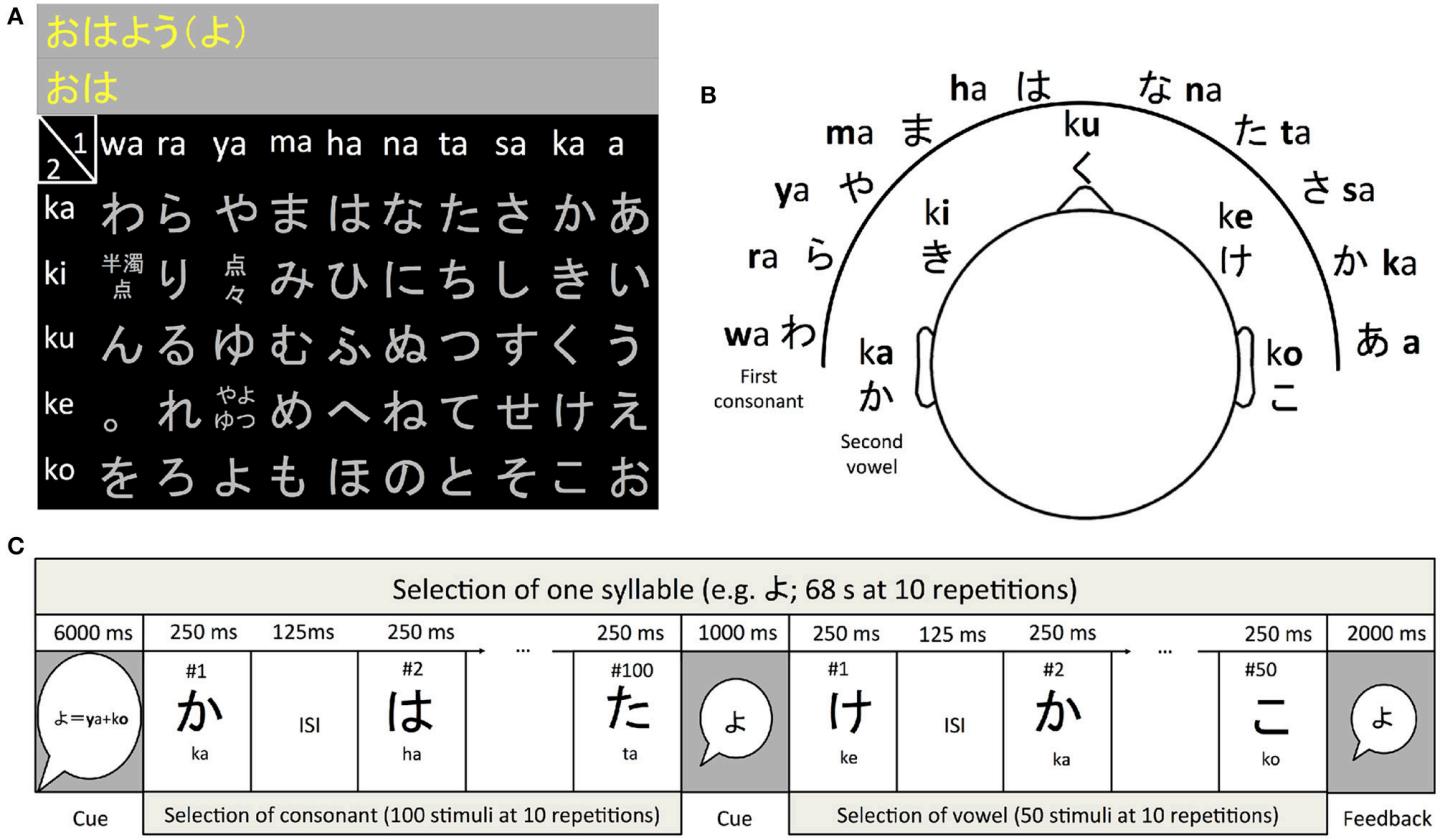
**The Hiragana speller used in this study**. The symbols are arranged in a 5 × 10 matrix **(A)**. In this design the user first heard the ten syllables ending with “a” from ten different directions **(B)**. These were used to select the consonant. After presentation of the stimuli used to select the consonant five syllables for selecting the vowel were presented **(C)**. Note that the actual stimulus duration varied due to the length of the sound files used for stimulation (see analysis of sound files in results section). The stimulus-onset-asynchrony of 375 ms was constant.

The modern Japanese Hiragana syllabary consists of 46 characters. Selecting from these characters in a stream based P300 approach with e.g., 10 stimulus repetitions would require 460 stimulus presentations. In P300 speller BCI this number is commonly reduced by arranging the symbols so that they can be selected in a matrix. To our advantage, the Hiragana syllabary is constructed around a 5 × 10 matrix called the “fifty sounds” which can be used to implement an intuitive hierarchical selection approach for an auditory P300 speller (see Figure 1). In this matrix the syllables are grouped according to the consonant they begin with and the vowel at the end. In our study, users first selected the consonant followed by the selection of the vowel (see Figure 1). The motivation for the design was to provide the users with a natural sequence of selection (all syllables in the Hiragana syllabary begin with a consonant) and minimize the number of different stimuli the user has to learn to recognize. In all studies the stimuli were recorded with a female voice. During the online experiment, feedback was displayed using the Latin alphabet.

### Participants

The study was performed with six healthy participants (5 male, average age 32.6 years) and one participant with SCI. The participants had no neurological signs and reported no hearing deficits. The end-user was male, 43 year old, diagnosed 23 years ago with a C3/C4 spine injury. The study was approved by the institutional ethics committee at the National Rehabilitation Center for Persons with Disabilities and all participants provided written informed consent according to institutional guidelines. All experiments were carried out in accordance with the approved guidelines.

### Procedure

#### Stimuli set design

In our current approach (see Figure 1) we propose the user will select the consonant first (a choice of one out of ten) and then the vowel (a choice of one out of five). Therefore, the participants always used the syllables wa, ra, ya, ma, ha, na, ta, sa, ka, and the vowel “a” for selection of the column of the matrix and ka, ki, ku, ke, and ko for selection of the vowel. We opted for this stimuli set for the following reasons. One, using a set of fixed stimuli (instead of one stimulus per target) eliminates the need to have a stimulus for all syllables (e.g., the “n” sound is difficult to ignore) and allows the introduction of special symbols such as the “.” and character modifiers such as the dakuten marker. And two, the user only has to learn to recognize 15 different stimuli instead of 46. Spatial cues were added to the stimuli according to the methods described in Käthner et al. (2013). Stimuli were presented using Etymotic ER4 MicroPro (Etymotic Research, Inc., USA) earphones.

The participants were asked to select 10 syllables offline (wa, mi, fu, te, o, wa, mi, fu, te, o) which were used to train the classifier and 15 syllables online with feedback (ra, yo, mu, he, ni, ke, su, chi, no, ha, hi, ma, ne, so, ku) to evaluate performance. Each stimulus was presented ten times for calibration and reduced to the number of repetitions needed to achieve 70% correct plus three repetitions (e.g., if 70% was reached after five repetitions during calibration we set the number of repetitions to eight during feedback). Online classification weights were obtained with stepwise linear discriminant analysis (SWLDA). Additionally, offline classification was performed with shrinkage linear discriminant analysis (SLDA). All participants took part in three sessions to determine if there are training effects (Baykara et al., 2016). In accordance with the training procedure proposed in Baykara et al. (2016) the number of repetitions was set based on the calibration data. If the offline accuracy using the calibration data surpassed 70% the number of repetitions was set to the number of repetitions this occurred at plus three (allowing for a maximum of 10 repetitions). The measurements were performed at the National Rehabilitation Center for Persons with Disabilities. Participants were provided with a visual support matrix that indicated what stimuli were needed to select the target syllable.

There was a pause of 8 s between syllables and a pause between consonant and vowel selection of 1 s. The stimulus onset asynchrony (SOA) was set to 375 ms. With 10 repetitions this results in a selection time of 1 min and 8 s. Note this is the upper bound and the final time varied due to the adaptation of the stimulus repetitions as part of the training procedure (see above).

#### Measurements with end-user with motor impairments

One end-user performed the tasks described in Section Stimuli Set Design. The measurements with the end-user were performed at the National Rehabilitation Center for Persons with Disabilities. The participant was provided with a visual support matrix that indicated what stimuli were needed to select the target syllable.

### Data acquisition

Electroencephalogram (EEG) data was recorded with a g.Tec g.USBamp (g.Tec GmbH, Austria) with a 0.1–30 Hz bandpass and 50 Hz notch filter with a sampling rate of 256 Hz. Sixteen active electrodes (g.Ladybird) were positioned in an electrode cap (g.Gamma) at AF7, FPz, AF8, F3, Fz, F4, C3, Cz, C4, CP3, CPz, CP4, P3, Pz, P4, and POz. Data recording, stimulus presentation and signal processing was controlled with BCI2000 (Schalk et al., 2004) on a Hewlett-Packard EliteBook 840 (HP Inc., USA) with a dual-core CPU (2.5 GHz), 8 GB RAM and a 64-bit Windows 7.

### Data analyses

We used SWLDA for training the online classifier and SLDA for offline classification. Comparisons and descriptions of classifiers can be found in Krusienski et al. (2006), Lotte et al. (2007), and Blankertz et al. (2011). For training the online classifier we used *p*-value thresholds of *p* < 0.1 for the forward step and *p* > 0.15 for the backward step and a maximum number of 60 features. Features were extracted from 0 to 1000 ms post-stimulus. Online no baseline correction was performed. A moving average filter with a width of 20 samples and subsampling to every 20th sample was applied to the date. During online classification weights were applied to the EEG features after each stimulus presentation and the resulting outputs were summed according to stimulus type. The vowel and consonant with the highest classifier score was selected. All participants received online feedback for runs three, four, and five (except participant two, session three, run three during which online feedback was not turned on by mistake).

Due to the high number of possible selections and the limited data we relied on leave one run (i.e., five stimulus selections) out cross-validation. We believe that in this case offline classification is preferable to reduce the strain on the participants by collecting a smaller calibration data set. Using cross-validation provides a more reliable estimate of the true classification rate by increasing the generalizability of the results. Offline classification was performed using SLDA because this method was shown to have a slight advantage over the commonly used SWLDA classifier (Blankertz et al., 2010). Gamma coefficients were determined empirically on the training data by using the value that yielded the best accuracy in the range from 0.01 to 0.1 (in 0.01 increments) and from 0.2 to 0.5 (in 0.1 increments).

To assess performance in terms of information transfer rate (ITR) we used the formula proposed in Wolpaw et al. (2002).

Offline analysis of the physiological data was performed under MATLAB using EEGLAB and self-written scripts (Delorme and Makeig, 2004). We distinguished two ERP components. An early negative component between 250 and 500 ms and a late positive component between 500 and 1000 ms on Cz. Trials were averaged using the data from individual sessions (three values per participant).

For statistical analyses we used a repeated measures analysis of variance (ANOVA). Analyses of the ITR, accuracy, and group level ERP amplitudes and latencies were performed using one value per participant (six) and session (three).

In an attempt to quantify the influence of the sound stimuli used on ERP latencies we took the absolute values of the sound files, smoothed them with a 1000-sample moving average window (the sampling frequency of the sound files was 44 kHz) and determined the latency of the amplitude maximum as well as the onset latency (the time period the sound file was below 1% of amplitude the peak amplitude). We then extracted the latencies for the early and the late ERP component for each stimulus file separately (fifteen in total; five for the rows and 10 for the columns). From the 15 individual ERP latencies we subtracted the mean latency of all 15 ERPs to normalize the data (so that there would be no effect of inter-individual differences and only the effect of the stimuli). We extracted the latencies for each session and averaged them. This resulted in 15 latencies (one for each stimulus) for each of the six participants. We then averaged again across participants and calculated Pearson's linear correlation coefficient with the latencies of the sound stimuli to determine if the sound file latency had an effect on ERP latency.

## Results

### BCI performance

All participants were asked to perform three sessions. In the following offline classification results using SLDA are reported. Vowel selection accuracy in session one was 61% (standard deviation (*SD*) 34, range 0–88, chance level 20%), in session two 65% (*SD* 35, range 8–96), and in session three 64% [*SD* 38, range 13–92; repeated measures ANOVA: *F*_(2, 10)_ = 0.29, *p* = 0.75]. Consonant selection accuracy in session one was 60% (*SD* 22, range 24–88, chance level 10%), in session two 69% (*SD* 29, range 28–92), and in session three 74% [*SD* 31, range 24–100; repeated measures ANOVA: *F*_(2, 10)_ = 4.38, *p* < 0.05]. Finally, Hiragana syllable selection accuracy in session one was 43% (*SD* 29, range 0–80), in session two 53% (*SD* 36, range 0–88), and in session three 57% [*SD* 39, range 7–92; repeated measures ANOVA: *F*_(2, 10)_ = 3.35, *p* = 0.08]. Online performance (Hiragana selection) of all participants was lower: In session one 37% (*SD* 25, range 0–60) in session two 41% (*SD* 34, range 0–80) and in session three 41% (*SD* 37%, range 0–87%).

**Figure 2:**
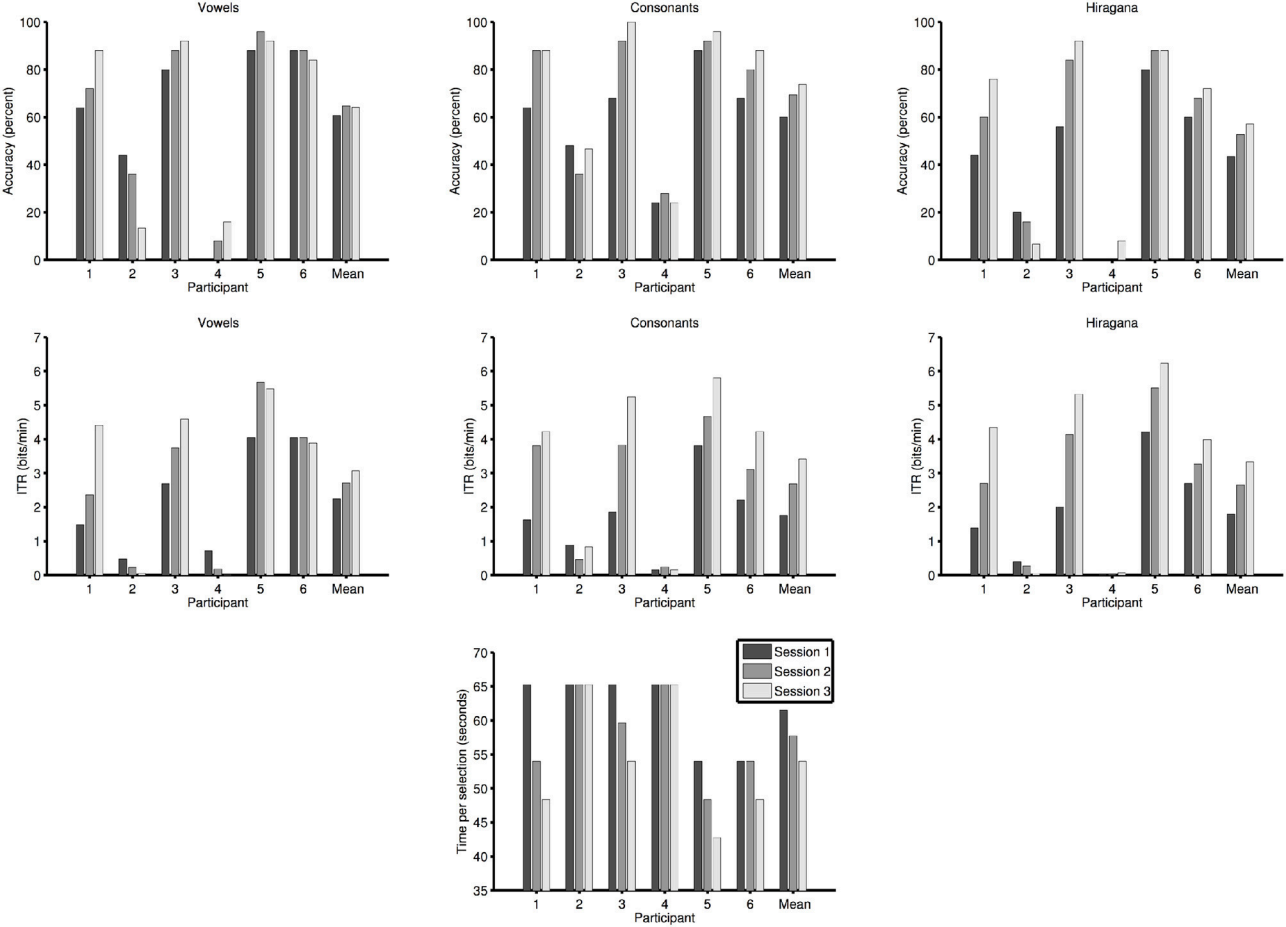
**Offline selection accuracy of the healthy participants**. Accuracies are shown separately for vowels, consonants, and Hiragana syllables (the latter being correct if both the correct vowel and consonant were selected). The accuracies do not take the decrease of selection times into account that occurred due to the reduction of stimulus repetitions. The second row shows the information transfer rates (ITRs) for vowel, consonant, and Hiragana selection (which conveys the most information per selection) of the healthy participants. The ITRs take the decrease of selection times into account that occurred due to the reduction of stimulus repetitions. The third row the time per selection needed by the healthy participants after the number of stimulus repetitions had been adjusted based on the performance in the calibration session.

In four out of six participants, there was a clear trend of increasing accuracy with session (see Figure 2 top row) and they reached accuracies above 70% in session three which would make the use of the BCI system for communication possible. Since we used an experimental design based on Baykara et al. (2016) that aims to maximize performance increase and not accuracy (the aim is to keep accuracy below 100% to avoid ceiling effects), ITR is more valid indicator of success than accuracy. The ITR of vowel selection in session one was 2.2 bits/min (*SD* 1.6, range 0.5–4.1), in session two 2.7 bits/min (*SD* 2.2, range 0.2–5.7), and in session three 3.1 bits/min [*SD* 2.4, range 0–5.5; repeated measures ANOVA: *F*_(2, 10)_ = 1.7, *p* = 0.24]. The ITR of consonant selection in session one was 1.8 bits/min (*SD* 1.3, range 0.2–3.8), in session two 2.7 bits/min (*SD* 1.9, range 0.2–4.8), and in session three 3.4 bits/min [*SD* 2.4, range 0.2–5.8; repeated measures ANOVA: *F*_(2, 10)_ = 7.4, *p* < 0.05]. Overall ITR of Hiragana selection in session one was 1.8 bits/min (*SD* 1.6, range 0–4.2), in session two 2.7 bits/min (*SD* 2.2, range 0–5.5), and in session three 3.3 bits/min [*SD* 2.7, range 0.1–6.2; repeated measures ANOVA: *F*_(2, 10)_ = 6.2, *p* < 0.05]. Thus, ITR increased for all stimuli sets from session-to-session and almost doubled for selection of Hiragana syllables. We observed increases in performance in four out of six participants for selection of consonants and Hiragana syllables (see Figure 2 middle row center and right). Participants five and six did not show increased performance in every session for vowel selection (see Figure 2 middle row left). Participants two and four showed no increase in performance in any category as could be expected based on the selection accuracies we obtained.

Figure 2 (bottom row) shows the decrease in selection time due to the reduction of the stimulus repetitions as part of the training procedure. All four participants that improved during the training (1, 3, 5, 6) needed a decreasing amount of time for one selection.

**Figure 3:**
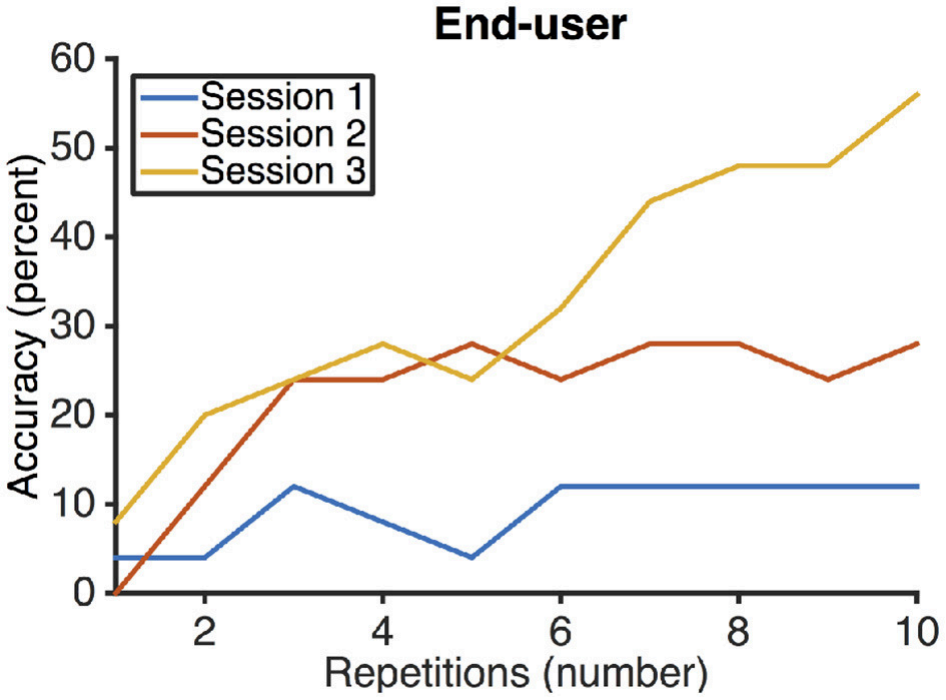
**Selection accuracies of the end-user participating in the study**. The end-user improved with each session.

The end-user achieved an accuracy of Hiragana selection of 12% in the first session, 28% in the second session, and 56% in the third session (see Figure 3). Vowel selection accuracy was 36, 44, and 64% in sessions one, two, and three. Consonant selection accuracy was 36, 56, and 72% in sessions one, two, and three. Corresponding ITR in sessions one, two, and three were 0.2 bits/min, 0.7 bits/min and 2 bits/min.

### Physiological data

Mean amplitudes of the early negative component decreased from −2.4 μV (*SD* 1.3) in session one, −3.2 μV (*SD* 1.2) in session two to −3.2 μV (*SD* 1.5) in session three. There was no main effect of session on amplitude [repeated measures ANOVA; *F*_(2, 10)_ = 2.03, *p* = 0.18]. Mean peak latencies remained fairly constant at 343 ms (*SD* 23) in session one, 373 ms (*SD* 67) in session two, and 346 ms (*SD* 33) in session three. Consequently, there was no main effect of session on latency [repeated measures ANOVA; *F*_(2, 10)_ = 1.18, *p* = 0.35]. Mean amplitudes of the late positive increased slightly for the healthy participants from 4.3 μV (*SD* 1.6) in session one, 4.6 μV (*SD* 2.5) in session two, and 4.9 μV (*SD* 2.2) in session three. There was no main effect of session on amplitude [repeated measures ANOVA; *F*_(2, 10)_ = 1.64, *p* = 0.25]. Mean peak latencies of the healthy participants decreased slightly from 706 ms (*SD* 113) in session one, 669 ms (*SD* 99) in session two, and 643 ms (*SD* 92) in session three. Again, there was no main effect of session [repeated measures ANOVA; *F*_(2, 10)_ = 2.98, *p* = 0.1].

**Figure 4:**
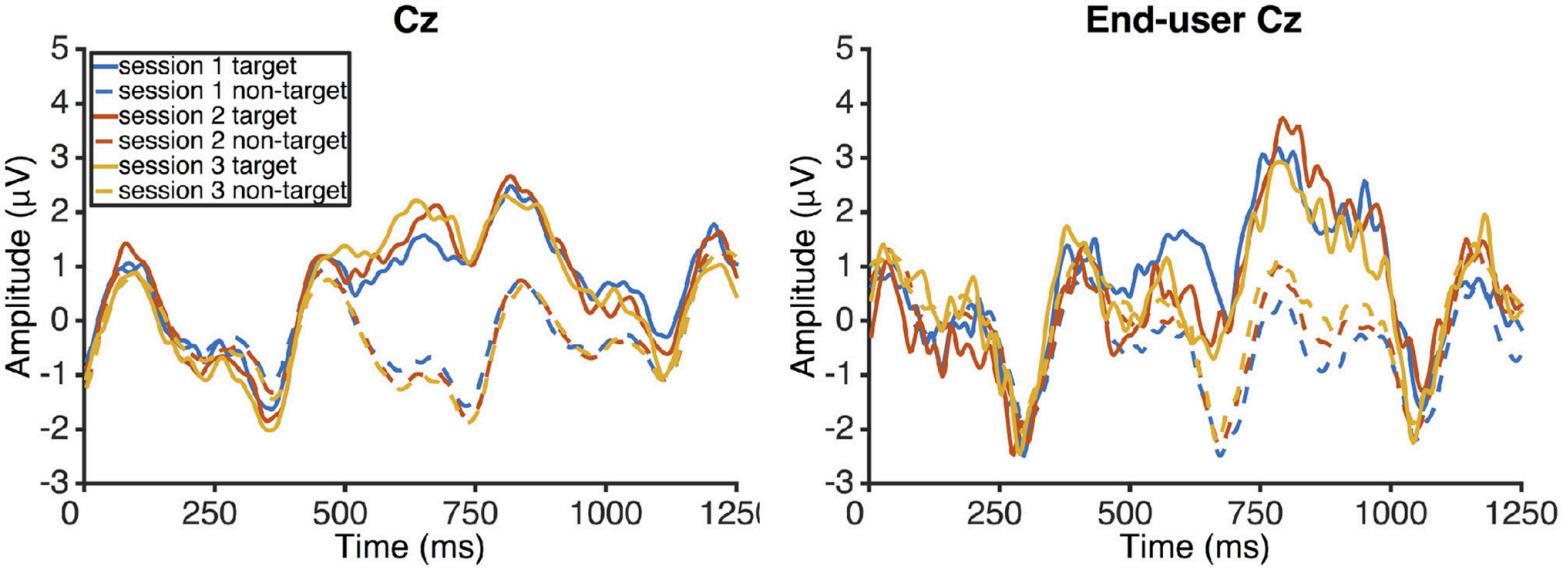
**ERP responses of the healthy participants in sessions one, two, and three (top left)**. The ERPs end-user one, who used the same system as the healthy controls, are shown top right. The continuous lines show target responses, the dashed lines non-target responses.

Mean amplitudes of the early negative component of the end-user were −2.8 μV (*SD* 1.5) at 306 ms (*SD* 11) in session one, −2.7 μV (*SD* 1.9) at 292 ms (*SD* 14) in session two, and −2.7 μV (*SD* 0.4) at 301 ms (*SD* 15) in session three. Mean late positive component amplitudes and latencies of end-user one were 4 μV (*SD* 1.1) with 810 ms (*SD* 59) latency in session one, 4.3 μV (*SD* 1.8) with 777 ms (*SD* 129) latency in session two, and 3.8 μV (*SD* 1.1) with 793 ms (*SD* 35) latency in session three. ERP from the study with healthy participants (left) and the end-user (right) are shown in Figure 4.

**Figure 5:**
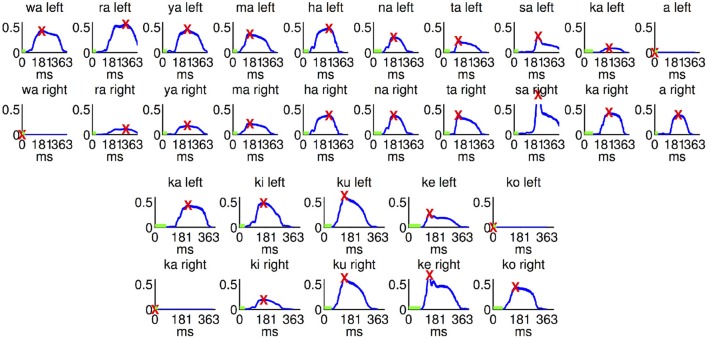
**The wave forms of the sound stimuli used in the current study**. We took the absolute values of the signals and smoothed them with a 1000 sample moving average. These curves are shown in blue for the right and left channel for each stimulus used. Maximum peaks are marked with a red “X.” Periods with less than 1% of the maximum amplitude are marked with a green bar. The column stimuli are shown in the top two rows and the row stimuli in the bottom two rows.

The wave forms of the sound stimuli are shown in Figure 5. The column selection sound stimuli had an average peak latency of 200 ms (*SD* 50). Mean peak latency of the row selection sound files was 163 ms (*SD* 35). The onset latency was 47 ms (*SD* 17) for the ten column selection stimuli and 62 ms (*SD* 19) for the five row selection stimuli. The correlation between the early and late ERP latencies of the all six participants with the latencies of the sound stimuli did not yield significant results. Upon exclusion of the two participants (number two and four) who did not generate ERP sufficient for BCI control the correlation between the latencies of the early ERP component and the latency of the peak of the sound stimuli became significant (*r* = 0.6, *p* < 0.05).

## Discussion

The participants controlled an auditory P300 BCI with a large number of possible selections (needed because of the large number of characters in the Japanese Hiragana syllabary). This is challenging because with the increasing number of possible selections a higher number of stimuli needs to be differentiated by the user (fifteen in the current study). Despite this challenge the four out of six able-bodied participants and the end-user were able to increase their performance with training. The four able-bodied participants that showed effects of training also reached the 70% accuracy needed for free communication.

### Selection speed and accuracy

Average performance was not as high as in e.g., Baykara et al. (2016) but four out of six participants reached accuracies high enough for free communication after three sessions with ITR between four and six bits/min. This is comparable to other implementations of auditory Hiragana spellers [e.g., three out of five participants achieved high accuracies in Chang et al. (2014)]. Comparable auditory spellers, for the Latin alphabet, with the aim to make the selection process deducible from the stimulus material have been more successful in terms of accuracy (Kleih et al., 2015). It should be noted that in the current study selections were made from 50 possible choices which is higher than previous studies (see Table 1 for a brief comparison) and making the task more difficult and the chance level lower (2% for Hiragana selection from the 5 × 10 matrix).

**Table 1:** **Four selected studies using auditory P300 for spelling**.

**Study**	**Number of choices**	**Participants with accuracy >70**	**Average accuracy**	**Symbol set**
Baykara et al., 2016	25	13 of 16	77	Latin alphabet excluding Z
Chang et al., 2014	25	0 of 5	40	Hiragana subset
Kleih et al., 2015	27	8 of 11	84	Latin alphabet and backspace
Current study	50	4 of 6	51	Hiragana syllabary

*Two studies use the Hiragana syllabary and two the Latin alphabet. The list is not exhaustive and serves to put the current study into context with approaches that enable a lower number of selections. The table shows the number of different possible selections, the number of participants (all were healthy volunteers) which reached 70% accuracy, the average accuracy, and which symbol set was used for spelling. The average accuracy given for the current study is averaged across all three sessions (43, 53, and 57% in the first, second, and third session respectively)*.

Across all participants performance (measured by ITR) increased with training for all three sub-tasks: Vowel selection, consonant selection, and Hiragana selection. This increase was significant for consonant and Hiragana selection. Accuracies of vowel selection were also lower than for consonant selection which is surprising because the chance level is higher for vowel selection. Thus, it may have been more difficult to attend the stimuli used for vowel selection.

An analysis of subject-wise performance showed that two participants were unable to control the BCI. Both participants performed better at selecting the consonants than at selecting the vowels as did the majority of the participants. Both participants could not explain why they were unable to perform the task. Neither were any technical issues apparent. Thus, it can only be assumed that the particular BCI paradigm described in the current publication is not suitable for these two participants but it is unclear why they were unable to control the system. Generally, there is a number of participants that is unable to control a particular BCI system. This lack of aptitude (of “BCI illiteracy”) may be caused by the inability to generate the physiological response required for control [e.g., the sensorimotor-rhythm (SMR) in Blankertz et al. (2010)] or performing the task according to instruction may have not the same effect as in high aptitude users (Halder et al., 2011). Other factors show to have an impact on BCI performance include empathy in motivation (Kleih et al., 2010; Kleih and Kübler, 2013). Interestingly, empathy seems to be inversely related to BCI performance. The response to an auditory oddball task may be an indicator of subsequent BCI performance (Halder et al., 2013).

Concerning the stimuli material feedback one possibility may be that a different voice for the Hiragana stimulus material may have made the selection easier for the participants (e.g., a male voice instead of a female voice). Since we used the five stimuli for vowel selection it is also a possibility that the two users may have performed better using only the stimuli for consonant selection (with which they performed much better than with the stimuli for vowel selection). It is interesting to note that average accuracies of vowel selection of the healthy participants were equal to consonant selection accuracy in session one (57%) and lower in sessions two and three (67% consonants vs. 62% vowels in session two and 74% consonants and 64% vowels in session three). Due to the higher chance level of one out of five targets, instead of one out of ten, accuracy should have been higher. Counteracting this effect is the observation that P300 amplitude increases with decreasing stimulus probability, which should increase classification rates (Duncan-Johnson and Donchin, 1977).

### Physiological responses

We analyzed the effect of the three session training conducted in the current study on two different ERP responses: an early negative response and a late positive response. The negative response showed a tendency to decrease in amplitude with training whereas latency was constant. The effect on amplitude (and latency) was not significant. The observed amplitudes of the late positive component increased slightly from 4.3 μV in session one to 4.9 μV in session three. This increase was also not significant on the group level. The amplitudes were considerably lower in the current study than in Baykara et al. (2016) (around 4.5 μV, as opposed to around 10 μV) but of similar magnitude to what was found in Chang et al. (2014), in which comparable stimulus material was used.

The latency showed a trend to decrease with training (from 706 to 643 ms) but this trend was also not significant (the repeated measures ANOVA had a *p*-value of 0.1). Generally, latencies of the observed later positive ERP components were higher than in studies using tones as stimuli (Baykara et al., 2016) but similar to what was observed in studies using words (Furdea et al., 2009). Increased latencies may be due to increased time until the participant recognizes the target stimuli due to the higher complexity of words and the increased stimulus duration. This is not a general rule though as some studies show lower latencies for word stimuli around 500 ms [value based on visual inspection of Figure 3 in Kleih et al. (2015)]. In the current study there was a trend that was not significant to decreasing latencies with training as in Baykara et al. (2016). In a study with mildly motor impaired end-users a significant decrease in P300 maximal peak latency could only be found for one out of five end-users (Halder et al., 2016).

### Influence of sound stimuli

In the current study the peak amplitude latencies were higher than we expected and we conducted an additional analysis of the sound files to find a possible explanation for this delay. A previous study showed the choice of the sound stimuli has a large influence on the communication speeds that can be achieved with an auditory P300 BCI speller (Halder et al., 2016). In the current study we investigated the influence of the latency of the onset and maximum amplitude of the sound stimuli with the latency of the ERP components. Kutas et al. (1977) showed that stimulus evaluation time may have an influence on P300 latency. Thus, the time point a sound stimulus in the current P300 BCI speller setup is recognized may influence the latency of the subsequent ERP. If the variation of the peak latency between different stimuli is to high this may have an adverse effect on classification accuracy. Out of eight different correlations that we investigated in this study (early and late ERP component latency × sound file onset and sound file peak × all participants and four participants that achieved control) only one was significant without correction (early ERP component latency with sound file peak and the data of four participants). For a conclusive investigation of this effect more participants and more trials with sound stimulus would be needed.

Concerning the choice of stimulus material two approaches have been used in the design of auditory P300 spellers. One approach is to use artificial stimuli that do not convey information about what the selection will be (Halder et al., 2010; Schreuder et al., 2010; Käthner et al., 2013). This approach has the advantage that the stimuli can be shorter and have less variation in stimulus evaluation time leading to less variance in ERP latency. The disadvantage is that coding larger matrices such as the Hiragana syllabary would require a substantial amount of memorization on behalf of the user or depend on visual support. The alternative approach is to use stimuli that encode what will be selected such as the current work or Kleih et al. (2015). The clear advantage is less workload for the user due to the explicit relationship between stimulus and target. The disadvantage is the variance of the stimulus material. Ideally, the intuitive stimuli could be modified to have identical stimulus evaluation times. One may speculate, that the training effects observed in this study and others were a consequence of adaptation of the user to the stimulus latency variance leading to decreased stimulus evaluation times and thus more consistent ERP latencies and increased classification accuracies.

### Future perspective

One alternative to an auditory P300 BCI would be a P300 BCI using visual stimuli without gaze dependence. Some evidence suggests that there is extensive brain atrophy especially in the CLIS in most brain regions except the occipital lobe (Nagao, 2013; Oyanagi et al., 2014). The same study found that visually evoked potential (VEP) are preserved. Thus, BCI using visual stimulation through closed eyelids (Lim et al., 2013; Hwang et al., 2015) and covert SSVEP paradigms may be a viable alternative (Lesenfants et al., 2014). Steady-state visual evoked potential (SSVEP) based BCI have been shown to be possible using stimulation frequencies above the visibility threshold, which may increase comfort of use (Sakurada et al., 2015).

Another alternative would be a BCI using tactile stimuli. This can be a hybrid in the form of auditory and tactile stimulation as shown in Rutkowski and Mori (2015) or only tactile stimulation (Brouwer and van Erp, 2010; Kaufmann et al., 2013). One potential disadvantage of tactile BCI may be the diminishing tactile sensitivity that was described in at least one case of a completely locked-in end-user with ALS (Murguialday et al., 2011).

In some cases of ALS in which the disease progressed very slowly, degeneration in the brain was confined to the motor system (Mochizuki et al., 2016). Users with this (rare) form of ALS may be able to successfully communicate using a variety of sensory pathways.

Current research has shown that training visuospatial attention with tasks such as video games leads to higher accuracies in covert attention tasks (Mack et al., 2016). Possibly, such training tasks would translate to performance in covert attention tasks such as auditory or tactile P300 spellers.

We hope that changes in the stimuli set, using more speakers for better spatial discriminability or using tactile instead of auditory stimuli will increase system usability to a level that will make it helpful for end-users with levels of paralysis ranging from light to complete.

## Conclusions

The results with healthy participants and end-user one are encouraging. We have shown that training increases BCI performance with a variety of user groups and stimulus material in the current work and in previous publications (Baykara et al., 2016; Halder et al., 2016). In particular, we would like to highlight the fact that the participants were able to make a reliable selection out of 50 possible choices.

## Author contributions

SH and KK: Designed the experiment; SH, KT, and HO: Prepared the experimental setup; SH, KT, and KU: Collected the data; SH and AO: Analyzed the data; SH and KK: Drafted the manuscript; SH, KT, HO, AO, KU, and KK: Revised the manuscript.

## Funding

The first author has received funding as International Research Fellow of the Japan Society for the Promotion of Science and the Alexander von Humboldt Foundation. This study was partly supported by a MHLW/AMED grant (BMI), a MEXT/AMED-SRPBS grant, and MEXT/JSPS grants (15H03126, 15H05880, 16K13113, and 16H05583).

### Conflict of interest statement

The authors declare that the research was conducted in the absence of any commercial or financial relationships that could be construed as a potential conflict of interest.
